# Supplementary material for: Antibodies against medically relevant arthropod-borne viruses in the ubiquitous African rodent Mastomys natalensis
Source: PLoS Negl Trop Dis. 2024 Sep 4;18(9):e0012233. doi: 10.1371/journal.pntd.0012233 (PMC11404846; doi:10.1371/journal.pntd.0012233)
Supplement: S1 File — (PDF) [file pntd.0012233.s001.pdf]

SOP-Luminex: Arbovirus antibody detection using Luminex technology in blood or serum

Contents

1. OBJECTIVE 1
2. DEFINITIONS 1
3. HYGIENE AND SECURITY 1
4. REAGENTS AND CONSUMABLES 2
4.1 REAGENTS 2
4.2 MATERIALS 2
4.3 REAGENTS AND BUFFER PREPARATIONS 2
5. PROCEDURE 3
5.1 KIT COVALENT COUPLING OF RECOMBINANT PROTEINS TO THE BEADS (±3H45MIN) 3
5.2 COUNTING ON A HEMACYTOMETER 4
5.3 PLATE PREPARATION 5
5.4 BEAD-BASED IMMUNOASSAY (FULL PLATE ±2H15MIN) 5
6. READING BIOPLEX 200 7
6.1 PREPARING LUMINEX MACHINE 7
6.2 CALIBRATING LUMINEX MACHINE 7
6.3 PLATE READING 7

1. Objective

To develop a multiplex immunoassay for *Flavi-* and *Togaviridae* exposure in wildlife samples. The test is used to increase the understanding of the sylvatic lifestyle of arboviruses and create a clear picture on which animal species are involved in maintaining arboviruses in Africa during epizootic periods. Identifying these animal hosts and reservoirs species will help to better predict, control and prevent arbovirus spillover and emergence of epidemics. This protocol is based on the combined protocols of Karen Kerkhof, Joachim Mariën, Martine Peeters, Ahidjo Ayouba; structured, adapted and optimized by Wim De Kesel.

2. Definitions

The Bio-Plex® 100/200 is designed for the use of MicroPlex® Carboxylated, SeroMAP™, MagPlex® and MagPlex®-TAGTM microspheres. The MAGPIX is designed for the use of MagPlex polystyrene paramagnetic microspheres (beads) as a substrate.

This protocol is designed for both readout systems for which the MagPlex® polystyrene paramagnetic microspheres (beads) are used as a substrate. The principle of this technique is based on coating different magnetic beads with a specific recombinant protein so when multiplexed in one assay the different beads can capture and quantify various antibodies in one blood sample. The magnetic beads will be kept in the wells by using a magnetic separator during the protocol.

3. Hygiene and security

Sodium Azide is a toxic reagent used as a preservative of the buffers used in this protocol. It may cause liver and kidney damage or can be fatal if inhaled, absorbed through skin or ingested. All remaining biological materials should be considered as being potentially infectious.

Laboratory personnel should always wear a laboratory coat, eye protection and latex or nitrile gloves when using Sodium Azide. After use 70% Ethanol must be used to decontaminate the work surface.

4. Reagents and consumables

4.1 Reagents

Table 1: *Overview of the reagents and the company*

Reagents	Reference number	Company
**NaH_2PO_4**	28 013.264	PROLABO
**$Na2HPO_4$**	S0876	Sigma-Aldrich
**NaCl**	27 810.295	PROLABO
**dPBS (500mL solution)**	14190-094	GIBCO
**dPBS (tablets)**	BR 0014G (LOT# 1262056)	Oxoid
**BSA**	A4503	Sigma-Aldrich
**FCS (fetal calf serum)**	A4766801	Thermo Fisher (-20°C freezer)
**EDC**	03449 (LOT# BCBF5908V)	Perbio Science / Thermofisher
**Sulfo-NHS**	56485 (LOT# BCBJ4456V)	Perbio Science / Thermofisher
**Tween (20)**	P7949	Sigma-Aldrich
**Sodium Azide**	S2002	Sigma-Aldrich
**Anti-Mouse IgG Biotin antibody goat**	ref B7022	Sigma-Aldrich
**Streptavidin-R-phycoerythrin (SAPE)**		Fisher Scientific/Life technologies
**Calibration kit for Bio-Plex 100/200**		
**Validation kit for Bio-Plex 100/200**		
**Sheath fluid Bioplex**		
**Magnetic COOH beads**	MagPlexTM MC100XX-01	Bio-Rad/Luminex

4.2 Materials

Table 2*: Overview of the materials and the company*

Materials	Company
**Micro-pipettes (10; 200; 1000 µl)**	Eppendorf
**Multichannel pipettes (300 µl)**	Eppendorf
**Pipette tips (10; 200; 1000 µl)**	Neptune
**Vortex**	
**Plate shaker at room temperature**	
**Rotating wheel (15-30rpm)**	
**Centrifuge up to ≥8000rpm**	
**Magnetic separator (1.5mL tubes)**	Luminex Corporation
**Magnetic separator (96-well plates)**	Luminex Corporation
**BioPlex 100/200 system**	Luminex Corporation
**96-well plates (flat bottom plates)**	Sigma-Aldrich

Extra materials
Plate sealers, Duran bottle, Eppendorf tubes, tinfoil, falcon Tubes (15 & 50mL), waste receptacle, reagent reservoirs, refrigerator (2-8°), freezer (-20 & -80°C), disposable gloves, lab coat, tube racks, etc.

4.3 Reagents and Buffer preparations

Table 3: *Preparation reagents and buffers*

Reagents	Buffer type	Conc	pH	Store	Preparation
**Sulfo-NHS (S-NHS)**	Coupling agent	50mg/mL		4°C	Dissolve 10mg vial of Sulfo-NHS into 200µL H_2O → **prepare immediately before use or make aliquots.**
**EDC**	Coupling agent	50mg/mL		-20°C	Dissolve 10mg vial of EDC into 200µL H_2O → **prepare immediately before use or make aliquots.**
**PBS1X**	PBS	0.01M	7.4	4°C	Dissolve 1 tablet in 500mL dH_2O
**PBS Hypertonic**	PBS Hypertonic	0.01M	7.4	4°C	0.8g of NaH_2PO_4 + 2.5g of $Na2HPO_4$ + 88g of NaCl in 1L of dH_2O

**PBS-TBN**	Dilution buffer		7.4	4°C	10g BSA + 2000µL Tween20 in 500mL PBS Hypertonic + 50mL FCS + 450mL dH_2O (50% PBS Hypertonic, 1% BSA, 0.2% Tween20, 5% FCS, 45% dH_2O) → **Filtered!**
**PBS-BSA**	Reading buffer		7.4	4°C	1g BSA filtrated 0.05g Sodium Azide in 100mL PBS (PBS, 1% BSA, 0.05% Sodium Azide(NaN_3))

5. Procedure

5.1 Kit covalent coupling of recombinant proteins to the beads

Covalent coupling to carboxylated paramagnetic microspheres/beads (MagPlex® microspheres, Luminex Corp., Austin, TX, USA) is performed at Room Temperature (18-25°C). Make sure the microspheres are **always protected from light**. Preselect your desires bead numbers and recombinant proteins. Bead coupling for scale **1 coupling (1.25*10^6)**, increased coupling can be done, in that case increase marked volumes proportionally. All necessary buffers are included in the kit, it is recommended to create a homemade storage buffer (Reading buffer) to save the kit buffer. **EDC and S-NHS are not included in the kit.**

*Table 4: Table with bead numbers for each protein, the desired coating concentration for 1 coupling (1.25*10^6 beads), protein stock concentration and the volume of the stock needed.*

Bead number	Protein	Company; reference protein	Desired concentration (µg/1.25*10^6 beads)	Stock concentration (µg/µL)	Volume needed (µL)
12	**ZIKV (NS1)**	Interchim; 40544-V07H	2	0.25	**8.00**
13	**DENV2 (NS1)**	BioRad; PIP048A	1	1.01	**0.99**
15	**CHIKV (E2)**	Prospecbio; CHI-003	2.5	0.90	**2.78**
20	**WNV (NS1)**	Sinobiological; 40346-V07H	2	0.15	**13.33**
21	**USUV (NS1)**	The native antigen company; Ab218552	2	0.49	**4.08**
25	**DENV1 (NS1)**	Prospecbio; DEN-004	2	1.0	**2.00**
26	**YFV (NS1)**	The native antigen company; YFV-NS1	1.5	0.89	**1.69**
33	**RVFV (NP)**	The native antigen company; REC31640	2.5	0.19	**13.16**
36	**DENV3 (NS1)**	The native antigen company; DENV3-NS1	2	0.84	**2.38**
39	**TBEV (NS1)**	The native antigen company; TBEV-NS1	2	0.524	**3.82**
42	**WSLV (NS1)**	The native antigen company; REC31698	2	0.80	**2.50**
48	**CCHFV (NP)**	The native antigen company; REC31639	4	0.37	**10.81**
51	**DENV4 (NS1)**	The native antigen company; DENV4-NS1	2	0.46	**4.35**
56	**MAYV (E2)**	The native antigen company; REC31644	1	0.16	**6.25**
61	**ONNV (E2)**	Interchim; B4TG40	2	1.0	**2.00**

1. Remove the kit from the refrigerator, to acclimatize to room temperature for **20-30 min**
2. Vortex the bead stock for **30 seconds**, sonicate for **30 sec**
3. Pipette 100µL of **bead stock solution** in a 1.5 mL "Protein LoBind" quality Eppendorf tube
4. Place tube in the magnetic holder for **1 min**
5. Eliminate the supernatant
6. Add 100µL of **Bead Wash Buffer** (provided in kit), vortex for **30 sec** and sonicate for **10 sec**
7. Place tube in magnetic holder for **1 min**
8. Eliminate the supernatant
9. Suspend the beads in 80µL of **Bead Activation Buffer** (provided in kit), vortex AND sonicate **30 sec**
10. Prepare **EDC** and **S-NHS** or thaw aliquots
11. Add 10µL of **EDC**, immediately vortex and add 10µL **S-NHS**, vortex **30 sec**

12. Place in rotator to incubate at room temperature in the dark for **20 min**
13. After 20 min, add 150µL of **PBS** (provided in kit), and vortex **10 sec**, place in magnetic holder **1 min**
14. Eliminate supernatant
15. Add 500µL of **PBS**, vortex **10 sec** and place in magnetic holder for **1 min**
16. Remove the supernatant (P1000, 500µL)
17. Re-suspend beads in 100µL of **PBS**, vortex for **30 sec** and sonicate for **15 sec**
18. Add the optimal amount of the recombinant protein to the activated beads (see following formula or table above)

Example equation when the stock conc. = 1.580 µg/µL; for a desired concentration of 8µg/1.25*10⁶; Vₐ= 5.06µL

$$Volume\ of\ antigen\ needed\ (V_a) = (\#\ of\ beads\ to\ couple)\frac{(Desired\ antigen\ conc.)}{(Stock\ antigen\ conc.)}$$

$$V_a = (1.25 * 10^6\ beads)\frac{(\#\mu g/1.25 * 10^6)}{(\#\mu g/\mu L)} = \frac{\#\mu g}{\#\mu g/\mu L}$$

19. Adjust the volume in the tube to 500µL (100µL PBS already in), add [400µL - V_a µL=…µL]* **PBS**
20. Incubate for **2 hours** at room temperature on a rotator in the dark
21. Place in magnetic holder for **1 min** and eliminate the supernatant
22. Wash beads with 500µL of **PBS**, vortex **10 sec** (do not sonicate!), place in magnetic holder for **1 min**
23. Eliminate the supernatant
24. Re-suspend the beads in 250µL of **Blocking Buffer** (provided in kit) and vortex for **15 sec**
25. Incubate **30 min** at room temperature, in rotator and in the dark
26. Place in magnetic holder for **1 min** and remove supernatant
27. Wash beads with 500µL of **Storage Buffer (homemade Reading buffer)**, vortex **10 sec** and place in magnetic holder for **2 min**
28. Remove supernatant and re-suspend beads in 150µL **Storage buffer** (provided in kit)

5.2 Counting on a Hemacytometer

Counting the beads on a Hemacytometer is performed to check if there is a loss of beads. The final concentration of beads can maximally be 8333 beads/µL (for a 1.25*10^6 bead coupling). That means that the counted amount of 10µL of a 1:10 dilution is optimally around 83 beads within one 4*4 corner grid.

1. Make a 1:10 dilution with dH_2O
   - 18 µL dH_2O
   - 2 µL coated bead solution
2. Count (in 4*4 corner grid) * dilution factor (10) *10 = # beads/µL ➔ C_i
   - Count (2-3) 4*4 corner grids and take average

5.3 Plate preparation

1. Prepare all the reagents
   - Each sample (1:200)
   - Include the positive pool dilution series
   - Include the negative samples
   - Include the blank samples (Dilution buffer)
2. Elution of DBS samples
   - 1 serobuvars dry blood spot (±0.5cmØ) in 200µL Dilution buffer overnight on shaker at 4°C results in a 1:100 dilution of the DBS ➔ if you elute in 400µL you get immediately a 1:200 dilution
3. Serum samples
   - Serum in stock is pure, for reading them it is also a 1:200 dilution, dilute with Dilution buffer
4. Add 50µL of the diluted samples in the appropriate wells
   - Vortex sample before adding
   - Pipette up & down
   - Cover plate with sealing tape and tinfoil, store in freezer (-20°C) **if not immediately used**
   - Label plate with name, date, and content

5.4 Bead-based immunoassay

1. Prepare all the reagents
2. Resuspend the coupled beads by vortexing for **30 sec**
3. Prepare the coupled beads by diluting to a concentration of 1000 beads/well (V_f = 25µL/well) in **Dilution buffer**
   - Example for 1 whole plate → 25µL/well * 100 wells = 2.5mL
   - C_f: Final concentration: 1000 beads/well = 40 beads/µL
   - V_f: Final volume: 25µL/well = 2500 µL/plate
   - C_i: Counted Beads conc. (Beads/ µL): see 5.2 Hemacytometer
   - $Volume\ of\ beads\ needed\ (V_i, in\ \mu L) = \frac{C_f * V_f}{C_i}$

   ⇨ Combine needed volume (Vi) of the different beads sets, add Dilution buffer until final volume (Vf) is reached (bead mix)

   Add 25µL (1000 beads*#bead sets) of the **bead mix** in the appropriate wells
4. Cover the plates with sealing tape and tinfoil to protect the beads against light
5. Incubate for **1 hour** on a plate shaker (600rpm) at room temperature
6. Wash the plate in the Washer with **Dilution buffer**
   - Prime washer with the **Dilution buffer** (check for bubbles in tubes, additional prime wash)
   - Choose protocol (*Run3: Lumin2*) and follow steps on machine
   - Insert plate (machine can skip empty columns but not empty rows, keep in mind)
   - "Cover" machine with foil to keep plate in dark, full plate takes about 7 min
   - Washer gives your plate back dry, all buffer liquid removed
7. Prepare the anti-host IgG-gamma chain specific biotynil antibody (500µg/mL, check with your product) by diluting the secondary anti-body to a concentration of 4µg/mL per well (V_f = 50µL/well) in **Dilution buffer**
   - C_f: Final concentration: 4µg/mL=0.004µg/µL
   - V_f: Final volume: 50µL/well → 5000 µL/plate
   - C_i: Initial concentration: 500µg/mL= 0.5µg/µL

- $Volume\ anti-host\ IgG\ stock\ (V_i, in\ \mu L) = \frac{C_f * V_f}{C_i}$
- Full plate : 96 wells ~ 100 wells : 40µL 2nd Ab in 4960µL Dilution buffer

Add 50µL of secondary antibody dilution in each well

8. Cover the plate with sealing tape and tinfoil to protect the beads from light
9. Incubate for **40 minutes** on a plate shaker (600rpm) at room temperature **(Warm up Luminex, 30min)**
10. Wash the plate in the Washer with **Dilution buffer**
    - Prime washer with the **Dilution buffer** (check for bubbles in tubes, additional prime wash)
    - Choose protocol and follow steps on machine
    - Insert plate (machine can skip empty columns but not empty rows, keep in mind)
    - "Cover" machine with foil to keep plate in dark, full plate takes about 7 min
    - Washer gives your plate back dry, all buffer liquid removed

11. Prepare the Streptavidin-PE (SAPE) solution by diluting the stock (1mg/mL) to a concentration of 1µg/mL (V_f= 50µL/well) in **Dilution buffer**. (Protect stock and solution from light!)
    - C_f: Final concentration: 1µg/mL=0.001µg/µL
    - V_f: Final volume: 50µL/well → 5000 µL/plate
    - C_i: Initial concentration: 1mg/mL= 1µg/µL
    - $Volume\ SAPE\ stock\ needed\ (V_i, in\ \mu L) = \frac{C_f * V_f}{C_i}$
    - Full plate : 96 wells ~ 100 wells : 5µL SAPE in 4995µL Dilution buffer

    Add 50µL of SAPE solution in each well

12. Cover the plate with sealing tape and tinfoil to protect the beads from light
13. Incubate for **10 minutes** on a plate shaker (400rpm) at room temperature **(Start-up & Calibrate Luminex)**
14. Wash the plate in the Washer with **!!! Reading buffer !!!**
    - Prime washer 2x with the **Reading buffer** (check for bubbles in tubes)
    - Choose protocol and follow steps on machine
    - Insert plate (machine can skip empty columns but not empty rows, keep in mind)
    - "Cover" machine with foil to keep plate in dark, full plate takes about 7 min
    - Washer gives your plate back dry, all buffer liquid removed
15. Add 150µL of **Reading buffer** to each well
16. Cover the plate with sealing tape and tinfoil to protect the beads from light (correctly label plate) and store at 4°C
17. Before plate reading
    - Place plate for **5 min** (same day) or **10 min** (following day) on a plate shaker (600rpm) before reading

6. Reading Bioplex 200

Remember that <u>sheath fluid is always filled</u> before running machine, and when shutting down machine that sheath fluid is at least half full and lid is loose. <u>Empty waste receptacle</u> before and after running machine.

6.1 Preparing Luminex machine

1. Turn on computer
2. Turn on the upper part of Luminex (Laser)
3. Turn on the lower part of the Luminex (Reader)
4. Launch the "Bioplex manager" application (desktop shortcut)
5. To warm up lasers: click on "Warm Up" (**30 min**) (located in the top tab)

6.2 Calibrating Luminex machine

1. Right panel in Bioplex application, "Quick Guide", click on "Start Up" & "Calibrate"
2. In the window that appears check that the indications describe the calibrators or if it matches the used calibrators, click on "Ok"
3. Fill the Bioplex MVC flat III plate with mQ water (fill ¾) in the indicated locations (see display, yellow flashing areas)
4. Vortex the calibration vials (red and green) for **30 sec**
5. Add <u>5 drops</u> of each calibration vial in the corresponding (colored) wells
6. Open the plate reader by clicking "eject/retract" in the Bioplex application
7. Place the plate correctly in the plate reader and click again "eject/retract"
8. Click "Start" to start calibration (**10 min**)
   Note: The calibration is valid if the temperature of the reading chamber has not changed more than ±2°C, beyond that re-calibration is required. If this is the case the machine detects it and locks it. A message appears on the screen, click "Ok" and re-calibrate.

6.3 Plate reading

9. Click "New protocol" in application
10. Select appropriate beads (MagPlex) and numbers
11. Select locations on the plate and fill in sample numbers according to location on the plate
    - Label all samples as "Samples X" and fill in the specific names in next tab
12. Limit reading to **"<u>45" seconds</u> ("Sample time out")** and check **<u>"Run at High RP1 Target"</u>**
13. Check if Gate settings (Advance settings) are limited by **<u>3000 and 30000</u> MFI values** (override gates)
14. Click start and wait (you can time how long machine takes to read 1 well and calculate when machine will be done)
15. Save output in folder "Wim Wildlife", send a copy of the data to wim.dekesel@uantwerpen.be
16. After reading click "Shut down" if finished (or perform wash step between plates with different proteins or different method)
    - Remove plate from machine ("eject/retract") (**<u>Keep plate, store in fridge, labelled!</u>**)
    - Shutting machine down by filling Bioplex MVC flat III plate with mQ water and bleach in corresponding location (takes **9 minutes**)
    - Rinse plate with tap water and tap dry and place back into plate slot
    - Empty waste receptacle (sink) and fill sheath fluid at least half and loosen lid
