# Supplementary material for: Antibodies against medically relevant arthropod-borne viruses in the ubiquitous African rodent Mastomys natalensis
Source: PLoS Negl Trop Dis. 2024 Sep 4;18(9):e0012233. doi: 10.1371/journal.pntd.0012233 (PMC11404846; doi:10.1371/journal.pntd.0012233)
Supplement: S2 File — (HTML) [file pntd.0012233.s002.html]

RMarkdown: Antibodies against medically relevant arthropod-borne viruses in the ubiquitous African rodent Mastomys natalensis


# RMarkdown: Antibodies against medically relevant arthropod-borne viruses in the ubiquitous African rodent Mastomys natalensis

#### De Kesel et al.

#### 2024-07-25

## Packages

```
library(tidyverse)
library(readxl)
library(changepoint)
library(ggpubr)
library(fitdistrplus)
library(ggplot2)
library(Matrix)
library(lme4)
library(emmeans)
library(multcomp)
library(gplots)
library(RColorBrewer)
library(Cairo)
library(psych)
library(dendextend)
library(gridExtra)
library(segmented)
library(strucchange)
library(ecp)
library(geomtextpath)
library(svglite)
library(binom)
library(DT)
```

## Data import

```
#Importing the Complete data set
CompleteData <-as.data.frame(read_excel("C:/Users/wimde/OneDrive - Universiteit Antwerpen/Data&Results/Arboviruses/Luminex_results/Mastomys/SerostatusMn500_800/DataPaper2024.xlsx", sheet = "CompleteData"))

#Filter the Complete data on beadset
CompleteData %>% filter(BeadSet == 1) -> BS1 #Extracting the the results of the first bead set (BS1)
CompleteData %>% filter(BeadSet == 2) -> BS2 #Extracting the the results of the second bead set (BS2)
```

## Transforming raw MFI data to units based on the dilution series as standard curve

```
#Simplifying all scripts which will select all MFI columns, by assigning the column names of interest to a vector name
ARBOname<-c("RVFV","YFV","ZIKV","DENV1","DENV2","DENV3","DENV4","USUV","WNV","TBEV","WSLV","CCHFV","CHIKV","MAYV","ONNV")

#Double loop for transforming the unknown MFI results of bead set 1 (BS1) to a unit measurement based on the positive dilution curve which was included in each experiment. Dilution series is 1:200, 1:2000, 1:20000 and 1:200000; corresponding units are 3125, 312.5, 31.25 and 3.125. ONNV is excluded because was not included in BS1
ExpCode1<-unique(BS1$Experiment) #List of the experiment codes
BS1_Units<-BS1 #Copy data into new data frame to store the unit values

#First loop, loops through each experiment and calculates the curve for each arbovirus according to the positive control dilution and transforms raw MFI to units
for(Exp in ExpCode1){ 
  #Filter the data for experiment and only the positive dilution series
  BS1 %>% filter(Experiment==Exp & Sample == "Positive") ->DT 
  
  DT<-as.data.frame(DT) #Store as data frame
  
  #Second loop, loops through each tested arbovirus excluding ONNV
  for(ARBOV in ARBOname[ARBOname!="ONNV"]){
    print(paste("Plotting dilution curve of experiment", Exp, "for", ARBOV))
    
    #y vector starting at 3125 every step divided by 10, until 3 values after 3125, log transformed
    y<-log(cumprod(c(3125,rep(1/10,3)))) 
    #Log transforming the data
    x<-log(DT[,ARBOV]) 
    PosDil <- data.frame(unit = y, MFI = x)
    
    #If an error occurs with the sigmoidal curve fitting use linear curve otherwise use sigmoidal
    result<-tryCatch({
      nls(MFI ~ SSlogis(unit, Asym, xmid, scal), 
          data = PosDil, 
          control = nls.control(minFactor = 1e-15, maxiter = 10000))
      }, error=function(e){
        print("try-error")
        })
    
    if(any(result=="try-error")){ 
      print("Performing alternative code")
      fit<-lm(y~x) #Fit is the fitted linear curve model
      
      #Curve of the fitted data
      plot(exp(x), exp(y), 
           pch = 16, main = paste("Linear Fit of", ARBOV, "for experiment", Exp), 
           xlab = "MFI", 
           ylab = "Units")
      abline(fit,col="red")
      
      #Calculate the unit values according to the predict function
      BS1_Units[BS1_Units$Experiment == Exp, ARBOV]<- 
        exp(coef(fit)[2] * log(BS1[BS1$Experiment == Exp, ARBOV]) + coef(fit)[1])
      
      }else{
        fit <- nls(MFI ~ SSlogis(unit, Asym, xmid, scal), 
                   data = PosDil, 
                   control = nls.control(minFactor = 1e-15, maxiter = 10000))
        
        PosDil$fitted <- predict(fit) #Fit is the fitted sigmoidal curve model
        #Curve of the fitted data
        curve<-ggplot(PosDil, aes(x = exp(fitted), y = exp(unit))) +
          geom_point() +
          labs(x = "MFI", y = "unit",title=paste(Exp,":",ARBOV,"positive dilution"))
        
        #A function to predict unit values based on MFI values according to the sigmoidal fit model
        predict_unit <- function(newMFI, fit) {
          xmid <- coef(fit)["xmid"]
          scal <- coef(fit)["scal"]
          Asym <- coef(fit)["Asym"]
          predicted_unit_values<-vector()
          for(i in 1:length(newMFI)){
            predicted_unit_values[i] <- if(is.nan(xmid - scal * log((Asym / newMFI[i]) - 1))){
              ReCal<-newMFI[i]-((newMFI[i]-Asym)+(newMFI[i]-Asym)^-0.1)
              xmid - scal * log((Asym / ReCal) - 1)
              } else{
                xmid - scal * log((Asym / newMFI[i]) - 1)
              }
            }
          return(predicted_unit_values)
        }
        
        #Create an example of the fitted curve
        additional_points <- data.frame(MFI = seq(1,max(PosDil$fitted),0.01), 
                                        unit = predict_unit((seq(1,max(PosDil$fitted),0.01)), fit))
        print(curve+geom_line(data = additional_points, 
                              aes(x = exp(MFI), 
                                  y = exp(unit)), 
                              color = "red", 
                              linewidth = 1))
        
        #Calculate the unit values according to the predict function
        BS1_Units[BS1_Units$Experiment == Exp, ARBOV] <- 
          exp(predict_unit(log(BS1[BS1$Experiment == Exp, ARBOV]), fit))
      }
  }
  }
```

```
#Double loop for transforming the unknown MFI results of bead set 2 (BS2) to a unit measurement based on the positive dilution curve which was included in each experiment. Same code as with BS1 only dilution series is different and ONNV is included in this set. Dilution series is 1:200, 1:1000, 1:5000, 1:25000, 1:125000 and 1:625000; corresponding units are 3125, 625, 125, 25, 5 and 1.
ExpCode2<-unique(BS2$Experiment) #List of the experiment codes
BS2_Units<-BS2 #Copy data into new data frame to store the unit values

#First loop, loops through each experiment and calculates the curve for each arbovirus according to the positive control dilution and transforms raw MFI to units
for(Exp in ExpCode2){ 
  #Filter the data for experiment and only the positive dilution series
  DT <- BS2 %>% filter(Experiment==Exp & Sample == "Positive") 
  DT <- as.data.frame(DT) #Store as data frame
  
  #Second loop, loops through each tested arbovirus
  for(ARBOV in ARBOname){
    print(paste("Plotting dilution curve of experiment", Exp, "for", ARBOV))
    
    #y vector starting at 3125 every step divided by 5, until 5 values after 3125, log transformed
    y <- log(cumprod(c(3125, rep(1/5, 5))))
    #Log transforming the data
    x <- log(DT[,ARBOV]) 
    PosDil <- data.frame(unit = y, MFI = x)
    
    #Creating the sigmoidal curve model
    fit <- nls(MFI ~ SSlogis(unit, Asym, xmid, scal), 
               data = PosDil, 
               control = nls.control(minFactor = 1e-15, maxiter = 10000))
    PosDil$fitted <- predict(fit)
    
    #Curve of the fitted data
    curve<-ggplot(PosDil, aes(x = exp(fitted), y = exp(unit))) +
      geom_point() +
      labs(x = "MFI", y = "log(unit)",title=paste(Exp, ":", ARBOV, "positive dilution"))

    #Create an example of the fitted curve
    additional_points <- data.frame(MFI = seq(1, max(PosDil$fitted), 0.01), 
                                    unit = predict_unit((seq(1, max(PosDil$fitted), 0.01)), fit))
    print(curve+geom_line(data = additional_points, 
                          aes(x = exp(MFI), y = exp(unit)), 
                          color = "red", 
                          linewidth = 1))
    
    #Calculate the unit values according to the predict function (same function as in BS1)
    BS2_Units[BS2_Units$Experiment == Exp, ARBOV] <- 
      exp(predict_unit(log(BS2[BS2$Experiment == Exp, ARBOV]), fit))
  }
  }
```

## Extracting all samples which are identical in both data sets from the unit data sets

```
#Find common samples using intersect
common_samples <- intersect(BS1_Units$UniqueSample, BS2_Units$UniqueSample)

#Extract rows from both data frames based on common samples
BS1_common <- BS1_Units[BS1_Units$UniqueSample %in% common_samples, ]
BS2_common <- BS2_Units[BS2_Units$UniqueSample %in% common_samples, ]

BS1_common <- BS1_common %>% group_by(UniqueSample) %>%
  summarise(ZIKV = mean(ZIKV,na.rm = TRUE),DENV2 = mean(DENV2, na.rm = TRUE),
            CHIKV = mean(CHIKV,na.rm = TRUE),WNV = mean(WNV, na.rm = TRUE),
            USUV = mean(USUV,na.rm = TRUE),DENV1 = mean(DENV1, na.rm = TRUE),
            YFV = mean(YFV,na.rm = TRUE),RVFV = mean(RVFV, na.rm = TRUE),
            DENV3 = mean(DENV3,na.rm = TRUE),TBEV = mean(TBEV, na.rm = TRUE),
            WSLV = mean(WSLV,na.rm = TRUE),CCHFV = mean(CCHFV, na.rm = TRUE),
            DENV4 = mean(DENV4,na.rm = TRUE),MAYV = mean(MAYV, na.rm = TRUE), ONNV = mean(ONNV, na.rm = TRUE))

BS2_common <- BS2_common %>% group_by(UniqueSample) %>%
  summarise(ZIKV = mean(ZIKV, na.rm = TRUE), DENV2 = mean(DENV2, na.rm = TRUE),
            CHIKV = mean(CHIKV, na.rm = TRUE), WNV = mean(WNV, na.rm = TRUE),
            USUV = mean(USUV, na.rm = TRUE), DENV1 = mean(DENV1, na.rm = TRUE),
            YFV = mean(YFV, na.rm = TRUE), RVFV = mean(RVFV, na.rm = TRUE),
            DENV3 = mean(DENV3, na.rm = TRUE), TBEV = mean(TBEV, na.rm = TRUE),
            WSLV = mean(WSLV, na.rm = TRUE), CCHFV = mean(CCHFV, na.rm = TRUE),
            DENV4 = mean(DENV4, na.rm = TRUE), MAYV = mean(MAYV, na.rm = TRUE), ONNV = mean(ONNV, na.rm = TRUE))

#Order the data based on unique ID
BS1_common <- as.data.frame(BS1_common[order(BS1_common$UniqueSample), ])
BS2_common <- as.data.frame(BS2_common[order(BS2_common$UniqueSample), ])
```

## Determining the formula for linear alignement of the common samples to transform the data of beadset 1 to fit with beadset 2

```
MODEL <- list() #Creating an empty list to store all the model values for each arbovirus
NewBS1_common <- BS1_common     #Creating a new data frame to store the recalculatedvalues

#For loop which calculates the intercepts, slopes, p-values and stores them in the corresponding data frames or lists
for(ARBOV in ARBOname[ARBOname!="ONNV"]){ 
  #model 1 is transforming BS1 to fit with BS2
  print(paste("Processing model 1 for", ARBOV))
  
  #creating model 1 BS2 is response, BS1 is explanatory
  model1 <- lm(log(BS2_common[, ARBOV]+1)~log(BS1_common[, ARBOV]+1)) 
  
  plot(log(BS1_common[,ARBOV]+1), log(BS2_common[, ARBOV]+1), 
       main=paste("Linear alignment model1 of common samples", ARBOV), 
       xlab="BS1", ylab="BS2")
  abline(model1, col="red")
  
  NewBS1_common[, ARBOV] <- exp(predict(model1, newdata=as.data.frame(log(BS1_common[, ARBOV]+1))))-1
  
  MODEL[[ARBOV]]<-model1
}
```

```
#Transforming all BS1 samples according to the calculated intercept and slopes from the common samples, to fit with BS2
TransfBS1 <- BS1_Units
for(ARBOV in ARBOname[ARBOname!="ONNV"]){ #for loop to recalculate and rewrite the new data set excluding ONNV
 
  intercept <- MODEL[[ARBOV]]$coefficients[1]
  slope <- MODEL[[ARBOV]]$coefficients[2]
  TransfBS1[, ARBOV] <- ifelse(exp(slope*log(BS1_Units[, ARBOV]+1)+intercept)-1 < 0, 0, 
                               exp(slope*log(BS1_Units[, ARBOV]+1)+intercept)-1)
}
```

## Merging the two data sets in units into one data frame for analysis (DFA)

```
#Merging the transformed BS1 unit data set with the BS2 unit data set
DFA <- merge(TransfBS1, BS2_Units, all=T) 

#Making age classes based on Weight using the quantiles. Four classes: Juvenile, Subadult, Subadult and Adult
DFA$Age <- cut(DFA$Weight, 
               breaks = quantile(DFA$Weight, probs = seq(0, 1, 1/3), na.rm=T), 
               labels = c("Juvenile","Subadult"," Adult"), include.lowest = TRUE)

#Excluding all data points with a dilution different than 1:200 (which is positive control dilution series)
DFA <- DFA %>% filter(Dilution == 200)
DFA <- as.data.frame(DFA)

#Removes all unknown samples were the weight or sex is NA
DFA <- DFA %>% filter(!(is.na(Weight)|is.na(Sex))|Sample !="Unknown")
```

## Cutoff based on Changepoint analysis

```
#An empty data frame to save the cutoff values, other cutoff values will also be saved in this data frame
CutOff<-data.frame(matrix(nrow=3,ncol=15)) #Empty data frame with 15 columns and 3 row
colnames(CutOff)<-ARBOname #Using same columns names as in other data sets
rownames(CutOff)<-c("CHP.m","CHP.v","CHP.mv") #Naming the row according to the cutoff calculation

#For loop to calculate the changepoint according to the different methodes
for (ARBOV in ARBOname) { 
  me<-sort(UnknownData[,ARBOV])
  position<-cpt.mean(me,penalty="None",method="AMOC",test.stat="Normal",class="FALSE")
  CutOff["CHP.m",ARBOV]<-me[position[[1]]]
  
  va<-sort(UnknownData[,ARBOV])
  position<-cpt.var(va,penalty="None",method="AMOC",test.stat="Normal",class="FALSE")
  CutOff["CHP.v",ARBOV]<-va[position[[1]]]
  
  meva<-sort(UnknownData[,ARBOV])
  position<-cpt.meanvar(meva,penalty="None",method="AMOC",test.stat="Normal",class="FALSE")
  CutOff["CHP.mv",ARBOV]<-meva[position[[1]]]
}
```

## Cutoff based on negative controls

```
#Adding a new row to cutoff table
CutOff<-rbind(CutOff,setNames(rep(NA,15),colnames(CutOff)))
rownames(CutOff)[nrow(CutOff)]<-"NegCtrl"

#Filtering the data for only the negative samples
DFA %>% filter(Sample == "Negative") -> NegData 
NegData<-as.data.frame (NegData)

#Negative cutoff is the mean plus 3 times the standard deviation
for(ARBOV in ARBOname){
  CutOff["NegCtrl",ARBOV]<- mean(NegData[,ARBOV],na.rm=T)+3*sd(NegData[,ARBOV],na.rm=T)
}
```

## Cutoff based on recaptured individuals

```
#Adding a new row to cutoff table
CutOff <- rbind(CutOff, setNames(rep(NA, 15), colnames(CutOff)))
rownames(CutOff)[nrow(CutOff)] <- "Recap"

UnknownData$Date<-as.Date(UnknownData$Date) #set to date format

#Selecting all individuals which were captured at least 3 times
{UnknownData %>% filter(Nr_Recap>=3) -> MultiCap

MultiCap$DSFC <- NA #Making a new column which will store the Days Since First Capture (DSFC)
MultiCap <- MultiCap[order(MultiCap$Date), ] #Ordering the data frame according to date

#For loop to calculate the DSFC and store in the corresponding column. 
for(i in sort(unique(MultiCap$UniqueID))){
MultiCap[MultiCap$UniqueID==i, "DSFC"] <- 
  as.numeric(MultiCap$Date[MultiCap$UniqueID==i] - min(MultiCap$Date[MultiCap$UniqueID==i]))
}

#Check for duplicates in dataframe
dup_rows <- duplicated(MultiCap$UniqueSample) | duplicated(MultiCap$UniqueSample, fromLast = TRUE)
  
#If there are duplicates, take the average of each value in every column
if (any(dup_rows)) {
  for (col_name in names(MultiCap)) {
    if (is.numeric(MultiCap[[col_name]])) {
    #Calculate the mean for each group of duplicate rows
    means <- tapply(MultiCap[dup_rows, col_name], MultiCap[dup_rows, "UniqueSample"], mean)
    #Replace the values in the original data frame with the calculated means
    MultiCap[dup_rows, col_name] <- means[as.character(MultiCap[dup_rows, "UniqueSample"])]
    }
  }
  #Keep only the first occurrence of each duplicate row
  MultiCap <- MultiCap[!dup_rows | !duplicated(MultiCap$UniqueSample), ]
}

#Large for loop which repeats this code for every tested arbovirus
for(ARBOV in ARBOname){
  #For loop to determine if we will use recaptured individual but only individuals that have not NA for the arbovirus
  for(i in sort(unique(MultiCap$UniqueID[!is.na(MultiCap[ARBOV])]))){
    #If the individuals maximum unit value is larger than 4 times the minimal value of that individual, then we keep the individual, otherwise it is removed.
    if((4*min(MultiCap[MultiCap$UniqueID==i, ARBOV], na.rm=T))>max(MultiCap[MultiCap$UniqueID==i, ARBOV], na.rm=T)
       ){
      MultiCap[MultiCap$UniqueID==i, ARBOV] <- NA
    }
    }
  
  #Making an empty data frame to store the data of the recaptured individuals after they are compensated for days
  Comp <- data.frame(Unit = character(),
                  Day = numeric(),
                  UniqueID = character(),
                  stringsAsFactors = FALSE)
  
  #For loop to compensate the days for each recaptured individual
  for(id in sort(unique(MultiCap$UniqueID[!is.na(MultiCap[ARBOV])]))){
    #Creating new vectors which represent the days and unit values
    DPI <- MultiCap[MultiCap$UniqueID == id& !is.na(MultiCap[ARBOV]), "DSFC"]
    AbUnit <- MultiCap[MultiCap$UniqueID == id & !is.na(MultiCap[ARBOV]), ARBOV]
  
    #Calculating day shift, setting peak unit value on day 30
    DayShift <- 30-DPI[which(AbUnit==max(AbUnit))]
    #Compensating the days with day shift
    DPI <- DPI+DayShift
    #Storing results as a new data frame and adding it to the compensated data frame
    new_data <- data.frame(Unit=AbUnit, Day=DPI, UniqueID=rep(id, length(AbUnit)))
    Comp <- rbind(Comp, new_data)
  }

  #Create ggplot plots with smoothed line of the data points (print if desired)
  ggplot(data=Comp, aes(x=Day, y=Unit, col=UniqueID))+
  geom_point()+
  geom_smooth(method = 'loess', se = F)+
  ggtitle(ARBOV)
  
  p<-ggplot(data=Comp, aes(x=Day, y=Unit))+
  geom_point()+
  geom_smooth(method = 'loess', se = TRUE)+
  ggtitle(ARBOV)
  
  #Extract the data from the drawn smoothed line, but only take the part between day 0 and 60
  smooth_data <- ggplot_build(p)$data[[2]]
  smooth_data <- subset(smooth_data, x>=0 & x<=60)
  
  #Find the maximum smoothed value
  CutOff["Recap",ARBOV] <- max(smooth_data$y)
}
}
```

Table with cutoff values for each arbovirus according to the
different methods

## Calculating the seroprevalence according to the five cutoffs

Barplots of seroprevalence according to the different cutoff values with
95% confidence interval and significant difference from ‘Recap’
seroprevalence

## Plotting all unit data in histograms with the cutoff values

Histograms of all data with cutoff levels

## Seroprevalence per arbovirus

Barplot with 95% confidence interval of seroprevalence for each tested
arbovirus

## Seroprevalence per viral family

```
## Overall arbovirus seroprevalence (positive for at least 1 arbovirus excluding ONNV): 
##            SeroPrevalence= 24.22%, 95% CI=21.89-26.66% 
##            Number of positives= 310, Total tested= 1280
```

```
## Overall flavivirus seorprevalence (positive for at least 1 flavivirus): 
##            SeroPrevalence= 20.16%, 95% CI= 17.99-22.46% 
##            Number of positives= 258, Total tested= 1280
```

```
## Overall alphavirus seroprevalence (positive for at least 1 alphavirus excluding ONNV): 
##            SeroPrevalence= 6.8%, 95% CI= 5.48-8.32% 
##            Number of positives= 87, Total tested= 1280
```

## Correlation on seroprevalence (measure for cross reactivity)

Table with arbovirus serostatus correlation (%)

Table with the significance of arbovirus serostatus correlation
(p-value)

## GLM seroprevalence data

```
#Glm model construction with sex, age and their interaction. Prevalence data family binomial. Post hoc test to pairwise compare estimates
PlotSeroSA2 <- list()
SPglmFull <- data.frame(matrix(nrow=15, ncol=9))     
colnames(SPglmFull) <- c("DevSex","ResDevSex","pSex","DevAge","ResDevAge","pAge","DevSA","ResDevSA","pSA")        
rownames(SPglmFull) <- ARBOname  

for(col in ARBOname){
  print(paste("### GLM prevalence of", col, " ###"))
  glm1 <- glm(PrevUnknown[[col]]~Sex*Age, data = PrevUnknown, family=binomial) #glm model
  AN <- anova(glm1, test='Chisq')
  SPglmFull[col, c(1,4,7)] <- round(AN$Deviance[-1], 2)
  SPglmFull[col, c(2,5,8)] <- round(AN$`Resid. Dev`[-1], 2)
  SPglmFull[col, c(3,6,9)] <- ifelse(AN$`Pr(>Chi)`[-1]<0.001, "<0.001", round(AN$`Pr(>Chi)`[-1], 3))
  
  #check if interactions are significant, depending on the result include or exclude interaction
  if (AN[4, 5]>0.1){
    glm1 <- glm(PrevUnknown[[col]]~Sex+Age, data = PrevUnknown, family=binomial)
    emmeans_obj <- emmeans(glm1, ~Sex+Age)
    AN <- anova(glm1, test='Chisq')
    inter <- "No"
    print("interaction no significant support")
  } else {
    glm1 <- glm(PrevUnknown[[col]]~Sex*Age, data = PrevUnknown, family=binomial)
    emmeans_obj <- emmeans(glm1, ~Sex*Age)
    AN <- anova(glm1, test='Chisq')
    inter <- "Yes"
    print("interaction significant support")
  }
  
  cld <- cld(emmeans_obj, type="respons", adjust="none") 
  cld <- cld[order(cld$Sex, cld$Age), ] #order the levels as they are in the dataframes
  
  print(as.data.frame(pairs(emmeans_obj, adjust="none"))[c(1, 2, 4, 7, 9, 10, 11, 14, 15), ])
  pairs <- as.data.frame(pairs(emmeans_obj, type="response", adjust="none"))
  PairList <- pairs[c(1, 2, 4, 7, 9, 10, 11, 14, 15), ]
  
  brackets <- data.frame(x=c(0.7, 0.7, 0.7, 1.7, 1.7, 1.0, 1.0, 2.0, 1.3),
    xend = c(1.7, 1.0, 1.3, 2.0, 2.3, 2.0, 1.3, 2.3, 2.3),
    y = c(max(cld[c(1:6),'asymp.UCL'][cld[c(1:6), 'asymp.UCL']<0.9])+max(cld[c(1:6), 'SE'])+0.08, 
        #juv female- juv male
        max(cld[c(1:2), 'asymp.UCL'][cld[c(1:2), 'asymp.UCL']<0.9])+max(cld[c(1:2), 'SE'])+0.01, 
        #juv-sub female
        max(cld[c(1:3), 'asymp.UCL'][cld[c(1:3), 'asymp.UCL']<0.9])+max(cld[c(1:3), 'SE'])+0.05, 
        #juv-adu female
        max(cld[c(4:5), 'asymp.UCL'][cld[c(4:5), 'asymp.UCL']<0.9])+max(cld[c(4:5), 'SE'])+0.025, 
        #juv-sub male
        max(cld[c(4:6), 'asymp.UCL'][cld[c(4:6), 'asymp.UCL']<0.9])+max(cld[c(4:6), 'SE'])+0.05, 
        #juv-adu male
        max(cld[c(1:6), 'asymp.UCL'][cld[c(1:6), 'asymp.UCL']<0.9])+max(cld[c(1:6), 'SE'])+0.08, 
        #F-M across/sub female - sub male
        max(cld[c(2:3), 'asymp.UCL'][cld[c(2:3), 'asymp.UCL']<0.9])+max(cld[c(2:3), 'SE'])+0.02,      
        #sub-adu female
        max(cld[c(5:6), 'asymp.UCL'][cld[c(5:6), 'asymp.UCL']<0.9])+max(cld[c(5:6), 'SE'])+0.025,      
        #sub-adu male
        max(cld[c(1:6), 'asymp.UCL'][cld[c(1:6), 'asymp.UCL']<0.9])+max(cld[c(1:6), 'SE'])+0.08)*100, 
        #adu female - adu male
        p = ifelse(PairList$p.value < 0.001, '***',
                 ifelse(PairList$p.value < 0.01, '**',
                 ifelse(PairList$p.value <= 0.1, '*', "NS"))),
                 textcol = c(NA, "black", "black", "black", "black", "black", "black", "black", NA),
                 extra = c("", "", "", "", "", "F-M:", "", "", ""),
                 vertAdj = c(0.6, 0.6, 0.6, 0.6, 0.6, -0.5, 0.6, 0.6, 0.6),
                 AdjYint = c(0.2, 0, 0, 0, 0, 1, 0, 0, 5))
  
  if(inter == "No") {
    PlotSeroSA2[[col]] <- PlotSeroSA[[col]]+
      geom_textsegment(data = subset(brackets, p!= "NS"), 
                       aes(x = x, xend = xend, y = y, yend=y, label = paste(extra, p), textcolour = textcol, vjust = vertAdj), 
                       size = 4, inherit.aes = F, linewidth = 0.4, lineend = "square") + 
      scale_y_continuous(limits = c(0, 40), breaks = seq(0, 40, 10))
    }else{
      PlotSeroSA2[[col]] <- PlotSeroSA[[col]]+
        geom_textsegment(data = subset(brackets, p!= "NS"), 
                         aes(x = x, xend = xend, y = (y+AdjYint), yend = (y+AdjYint), label=p), 
                         vjust=0.6, size=4 ,inherit.aes = F, linewidth=0.4, lineend="square") +
        scale_y_continuous(limits = c(0, 40), breaks = seq(0, 40, 10))
    }
  }

(Fullplot <- ggarrange(plotlist = PlotSeroSA2, ncol = 3, nrow = 5, common.legend = TRUE, legend = "bottom"))
```

Barplot with 95% confidence interval of seroprevalence for each tested
arbovirus according to age and sex with statistical significant
differences

```
{tiff("fullplot.tif", width = 2250, height = 2625, res = 300)
ggarrange(plotlist = PlotSeroSA2, ncol = 3, nrow = 5, common.legend = TRUE, legend = "bottom")
dev.off()}
ggsave("Fullplot.tiff", Fullplot, width = 1400, height = 1800, dpi = 200, units = "px")
```
