# Supplementary material for: Antibodies against medically relevant arthropod-borne viruses in the ubiquitous African rodent Mastomys natalensis
Source: PLoS Negl Trop Dis. 2024 Sep 4;18(9):e0012233. doi: 10.1371/journal.pntd.0012233 (PMC11404846; doi:10.1371/journal.pntd.0012233)
Supplement: Attachment — Submitted filename: Rebuttal letter PNTD_De Kesel et al..docx [file pntd.0012233.s007.docx]

Rebuttal letter PNTD-D-24-00720 – De Kesel et al.

Dear Editor,

Dr. Wang,

We appreciate the valuable and overall positive feedback from the referees on our manuscript entitled “Antibodies against medically relevant arthropod-borne viruses in the ubiquitous African rodent Mastomys natalensis” (PNTD-D-24-00720). We have carefully considered the points raised and refer for a detailed point-by-point response in the letter below.

With this letter, we now submit two versions (with and without track changes) of the revised manuscript addressing all the comments raised by the referees and hope that our manuscript is now acceptable for publication in PLOS NTD. We additionally added our lab protocol, detailed statistical analysis and raw data as extra supplementary information.

Sincere regards,

Wim De Kesel

Kevin Ariën

**Point-by-point response**

Reviewer's Responses to Questions

Methods

-Are the objectives of the study clearly articulated with a clear testable hypothesis stated? -Is the study design appropriate to address the stated objectives? -Is the population clearly described and appropriate for the hypothesis being tested? -Is the sample size sufficient to ensure adequate power to address the hypothesis being tested? -Were correct statistical analysis used to support conclusions? -Are there concerns about ethical or regulatory requirements being met?

**Reviewer #1:** -Are the objectives of the study clearly articulated with a clear testable hypothesis stated? Yes -Is the study design appropriate to address the stated objectives? Yes -Is the population clearly described and appropriate for the hypothesis being tested? Yes -Is the sample size sufficient to ensure adequate power to address the hypothesis being tested? Yes -Were correct statistical analysis used to support conclusions? Yes -Are there concerns about ethical or regulatory requirements being met? No

**Reviewer #2:** (No Response)

We appreciate that the reviewers are positive about the applied methodology.

--------------------

Results

-Does the analysis presented match the analysis plan? -Are the results clearly and completely presented? -Are the figures (Tables, Images) of sufficient quality for clarity?

**Reviewer #1:** The analysis presented match the analysis plan and results are clearly presented using good quality figures.

**Reviewer #2:** 1. Page 18, line 348 - Please correct grammar.

We have adapted line 357 (were 🡪 where), as requested by reviewer #2.

2. Page 23, line 453-458 - The authors showed that the DBS samples were cross-reactive with different viral proteins like NS1 of DENV and E2 of CHIKV, and they discussed the cross-reactivity to between different two proteins. So to well understand that point, please indicate the amino acid homology rate between NS1 and E2 proteins.

Most scientific literature focuses on the homology within the same family of viruses rather than between families. For example, high homology is noted between NS1 proteins of different flaviviruses (e.g., dengue, Zika) or E2 proteins of different alphaviruses (e.g., chikungunya, sindbis). Given their different biological functions, these proteins are not expected to have significant homology. The evolutionary distance between the *Flaviviridae* and *Togaviridae* families also supports a low similarity between these proteins. Based on their functional and structural divergence, the homology rate between flavivirus NS1 and alphavirus E2 proteins is expected to be extremely low. Comparing the amino acid sequences using the NCBI BLAST protein tool shows that there is no significant similarity found between the NS1 protein of DENV and E2 protein of CHIKV. Additionally, aligning the amino acid sequences in Geneious prime (i.e. Geneious alignment, MUSCLE alignment and Clustal Omega) results in a maximum homology rate of 12.6%.

We added the following sentence in line 474-476: “The homology rate between the two proteins is also less then 13% according to the amino acid alignment using Geneious Prime.”

3. Common experimental mouse has different sensitivity to viral infections between sex. Do authors think the sensitivity affect the seropositivity in your results?

Immune responses to infections have indeed been shown to be different between sexes, not only within rodent subjects but also in other vertebrates such as birds, fish and also humans. We therefore think that the sensitivity of each sex could indeed influence the seroprevalence. However, since the serostatus of each animal is determined on a cutoff value based on all the tested samples and the number of females and males is almost the same (660 females and 620 males) we considered this effect of sensitivity difference to be insignificant. If the sex ratio in the tested sample set would be different from 50% the results would be biased towards the sex with the larger ratio.

We added the following sentences in line 492-495: “These inherent sex differences in sensitivity to infections could influence the seroprevalence, where the calculated cutoff value could be an over or under estimation for a particular sex. However, since our sample size of males (N=620) and females (N=660) is approximately the same this influence was considered to be insignificant.”

4. Authors showed the seroprevalence of Arbovirus you listed. Are there reports that those viruses experimentally infect to mouse, and induced antibodies to NS1 or E2 proteins?

Several experimental mouse models exist to study arbovirus infections and the pathologies caused by these viruses. Depending on the type of studies, immunocompetent and immunocompromised mouse models are used, such as AG129, IFN-alpha/beta receptor or IFNAR-/- mice for dengue and Zika, C57BL/6 and BALB/c mice to study joint inflammation with CHIKV and RRV infection. Similar models exist to study the pathogenesis caused by CCHFV and RVFV. Vaccine studies that have used NS1 and E2 proteins in different mice models have shown that they indeed elicit an antibody response and in some cases protect the subject against a lethal dose of the infecting virus.

- Bailey, M. J., Broecker, F., Duehr, J., Arumemi, F., Krammer, F., Palese, P., & Tan, G. S. (2019). Antibodies elicited by an NS1-based vaccine protect mice against Zika virus. MBio, 10(2), 10-1128.
- Li, Y., Counor, D., Lu, P., Duong, V., Yu, Y., & Deubel, V. (2012). Protective immunity to Japanese encephalitis virus associated with anti-NS1 antibodies in a mouse model. Virology journal, 9, 1-13.
- Brault, A. C., Domi, A., McDonald, E. M., Talmi-Frank, D., McCurley, N., Basu, R., ... & Guirakhoo, F. (2017). A Zika vaccine targeting NS1 protein protects immunocompetent adult mice in a lethal challenge model. Scientific reports, 7(1), 14769.
- Weber, C., Büchner, S. M., & Schnierle, B. S. (2015). A small antigenic determinant of the Chikungunya virus E2 protein is sufficient to induce neutralizing antibodies which are partially protective in mice. PLoS neglected tropical diseases, 9(4), e0003684.
- Segato-Vendrameto, C. Z., Zanluca, C., Zucoloto, A. Z., Zaninelli, T. H., Bertozzi, M. M., Saraiva-Santos, T., ... & Verri, W. A. (2023). Chikungunya virus and its envelope protein E2 induce hyperalgesia in mice: Inhibition by anti-E2 monoclonal antibodies and by targeting TRPV1. Cells, 12(4), 556.
- Lum, F. M., Teo, T. H., Lee, W. W., Kam, Y. W., Rénia, L., & Ng, L. F. (2013). An essential role of antibodies in the control of Chikungunya virus infection. The Journal of Immunology, 190(12), 6295-6302.

5. Please correct Fig 2, especially Zika virus. Authors Zika virus belongs to Togaviridae. This is not correct.x

We had noticed ourselves this mistake shortly after submission of the manuscript and have now corrected Fig. 2 background color of ZIKV into *Flaviviridae*.

6. How is the sensitivity of this high-throughput multiplex immune assay?

In the absence of a realistic panel of negative control and naturally infected positive control samples it is difficult to estimate the true sensitivity of our assay. The sensitivity, and also the specificity, of this assay is for all tested arboviruses almost 100%. This is very unrealistic and can be explained by the used control samples. The used positive control samples, necessary to calculate the sensitivity, where created by inoculating the mice with an unnatural amount of pure viral protein. This was done to create control samples to test the success of the bead coating and to allow for a validation between assay runs. These control samples have such a high antibody titer that they would always test positive according to the cutoff value. Therefore the assay always identifies them as true positives. For the specificity, all negative controls originated from an inhouse breeding colony which has never been exposed to the natural environment. Therefore these samples are useful as negative controls to check whether there is contamination during each run. They are unfortunately not suitable to calculate the specificity since they do not represent realistic negative samples and the assay will always classify them as negative.

We altered the following sentences to clarify the sensitivity and specificity of the assay:

- Line 391-393: “The lack of realistic natural positive controls limits the possibility to calculate the assay’s sensitivity and determine a true cutoff, we therefore used recaptured seroconverted individuals to determine a cutoff value.”
- Line 405-407: “This can be explained by the fact that the negative control samples originate from a breeding colony and could thus also not be used to determine the assay’s specificity.”

--------------------

Conclusions

-Are the conclusions supported by the data presented? -Are the limitations of analysis clearly described? -Do the authors discuss how these data can be helpful to advance our understanding of the topic under study? -Is public health relevance addressed?

**Reviewer #1:** The authors conclude that *M. natalensis* play an important role in sustaining arbovirus transmission and this is supported by the data presented in the results (an overall seroprevalence of 24%). The paper clearly stated the limitations in their analysis such as using negative control from a breeding colony that could potentially over/under-estimate the sero-prevalence.

Do the authors discuss how these data can be helpful to advance our understanding of the topic under study? Yes

Is public health relevance addressed? Yes

**Reviewer #2:** (No Response)

We appreciate that the reviewers are supportive on the presented data and conclusions.

--------------------

Editorial and Data Presentation Modifications? Use this section for editorial suggestions as well as relatively minor modifications of existing data that would enhance clarity. If the only modifications needed are minor and/or editorial, you may wish to recommend “Minor Revision” or “Accept”.

**Reviewer #1:** Though the paper is well-written i think some background on the multiplex immunoassay will help to understand why the authors chose this method.

The method section is well-written however it would read better if the authors could consider adding subtitles to subsections e.g. Screening for IgG antibodies to arboviruses, recombinant arbovirus proteins etc

More details on beads coupling (if it was done)

We have added a sentence on the strengths of bead-based multiplex assays on line 376-382: “Besides the high throughput and multiplexing possibility, bead-based luminex assays come with several other advantages such as the small sample volumes required for testing, the compatibility with diverse sample types, incl. eluted DBS, the cost-effectiveness with lower reagent cost and reduced labor time, a broad dynamic range for accurate quantification, a reduced variability as a result of running multiple analytes in a single assay, and the versatility and flexibility of customizable panels.”

We have added subtitles in the methods section, as suggested by reviewer #1.

The bead coupling strategy is detailed in lines 162-166 and can also be found in the supplementary lab protocol.

**Reviewer #2:** (No Response)

--------------------

Summary and General Comments

Use this section to provide overall comments, discuss strengths/weaknesses of the study, novelty, significance, general execution and scholarship. You may also include additional comments for the author, including concerns about dual publication, research ethics, or publication ethics. If requesting major revision, please articulate the new experiments that are needed.

**Reviewer #1:** De Kesel and co-authors present an interesting study that address a key gap in the existing knowledge of arboviral diseases transmission dynamics. The authors hypothesize that rodents specifically *M. natalensis* may serve as amplifying hosts of arboviruses. To this end, they screened blood samples from *M. natalensis* individuals for antibodies against 15 medically important arboviruses and reported an overall arbovirus seroprevalence of 24% suggesting that *M. natalensis* plays a role in the transmission of multiple arboviruses. The main strength of this paper is that the authors chose a perfect rodent species to study arboviruses transmission dynamics. Besides being a reservoir host of numerous pathogens and also the most abundant rodent species in SSA, *M.* *natalensis* lives in close proximity to humans increasing the risk of pathogen spillover to humans. As such, the paper identifies rodents as potential sources of arbovirus infections that pose a threat to human health. One of the weaknesses or rather a limitation is that the authors used negative controls obtained from a breeding colony instead of natural positive controls which could have affected the cut-off values. I enjoyed reading this manuscript and i think the paper is well written. However, some background information on the immunoassay used is missing and the analysis, set up and protocol in the methods section could be structured into subsections with subtitles.

**Reviewer #2:** The authors developed a high-throughput multiplex immune assay using Luminex technology. The system employed the viral proteins such as NS1 protein for Flaviviridae and E2 protein for Togaviridae to evaluate the seroprevalence for 15 species of arboviruses in multimammate mouse and *Mastomys* *natalensis* in Tanzania. They showed the these African small mammals had highly seropositive rate to several arboviruses, such as Dengue virus type 2 (8.44%), Usutu virus (4.61%), Chikungunya virus (6.17%), and etc. Moreover, they presented the difference in seropositivity between sex or age. These results suggest that these small mammals play a role of transmission of arboviruses in African continent. This work is necessary to understand the viral circulations. This accumulates our knowledge to maintain public health.

If the authors continue this study, I'd like to try to isolate the arboviruses from African small animals.

We are thankful for this overall very positive feedback and supportive comments by both reviewers on the presented data and conclusions.

**PLOS Neglected Tropical Diseases Technical Check requests for your submission PNTD-D-24-00720R1 - [EMID:be91ca6472e80c69]**
1) Please upload the separate Supplementary files in numerical order

Supplementary files are now uploaded in numerical and chronological order as they are mentioned in manuscript.

2) Please ensure that the CRediT author contributions listed for every co-author are completed accurately and in full.
At this stage, the following Authors/Authors require contributions: Wim De Kesel, Bram Vanden Broecke, Benny Borremans, Léa Fourchault, Elisabeth Willems, Ann Ceulemans, Christopher Sabuni, Apia Massawe, Rhodes H Makundi, Herwig Leirs, Martine Peeters, Erik Verheyen, Sophie Gryseels, Joachim Mariën, and Kevin K Ariën. Please ensure that the full contributions of each author are acknowledged in the "Add/Edit/Remove Authors" section of our submission form.
The list of CRediT author contributions may be found here: <https://eur01.safelinks.protection.outlook.com/?url=https%3A%2F%2Fjournals.plos.org%2Fplosntds%2Fs%2Fauthorship%23loc-author-contributions&data=05%7C02%7Cwim.dekesel%40uantwerpen.be%7Cd8e5ebfb398949f19e4308dcb08f7f8d%7C792e08fb2d544a8eaf72202548136ef6%7C0%7C0%7C638579377569304348%7CUnknown%7CTWFpbGZsb3d8eyJWIjoiMC4wLjAwMDAiLCJQIjoiV2luMzIiLCJBTiI6Ik1haWwiLCJXVCI6Mn0%3D%7C0%7C%7C%7C&sdata=sM%2FaiCDUMHNekNWTnJSsatBpX46Qxm0FpWmg0SoTc44%3D&reserved=0>

The contributions for all authors is added.

3) We do not publish any copyright or trademark symbols that usually accompany proprietary names, eg ©,  ®, or TM  (e.g. next to drug or reagent names). Therefore please remove all instances of trademark/copyright symbols throughout the text, including:  MagPlex® on line no 165, and Quil-A® on line no 174.

The copyright and or trademark symbols are removed from the manuscript.

4) Please insert an Ethics Statement at the beginning of your Methods section, under a subheading 'Ethics Statement'. It must include:
i) The full name(s) of the Institutional Review Board(s) or Ethics Committee(s)
ii) The approval number(s), or a statement that approval was granted by the named board(s).

The ethics statement was mentioned in the acknowledgements and has been moved to a new subheading “ Ethics statement” in the methods section.

5) Please upload a copy of Figure 1 to 3 which you refer to in your text on page on the manuscript file. Or, if the figure is no longer to be included as part of the submission please remove all reference to it within the text.

All figures are now attached as files.

6) Some material included in your submission may be copyrighted. According to PLOSu2019s copyright policy, authors who use figures or other material (e.g., graphics, clipart, maps) from another author or copyright holder must demonstrate or obtain permission to publish this material under the Creative Commons Attribution 4.0 International (CC BY 4.0) License used by PLOS journals. Please closely review the details of PLOSu2019s copyright requirements here: PLOS Licenses and Copyright. If you need to request permissions from a copyright holder, you may use PLOS's Copyright Content Permission form.
Please respond directly to this email and provide any known details concerning your material's license terms and permissions required for reuse, even if you have not yet obtained copyright permissions or are unsure of your material's copyright compatibility. Once you have responded and addressed all other outstanding technical requirements, you may resubmit your manuscript within Editorial Manager.
Potential Copyright Issues:

i) Figure 1. Please (a) provide a direct link to the base layer of the map (i.e., the country or region border shape) and ensure this is also included in the figure legend; and (b) provide a link to the terms of use / license information for the base layer image or shapefile. We cannot publish proprietary or copyrighted maps (e.g. Google Maps, Mapquest) and the terms of use for your map base layer must be compatible with our CC BY 4.0 license.

Figure 1 originates from the website <https://d-maps.com/index.php?lang=en>, the base layers for the figure are <https://d-maps.com/carte.php?num_car=736&lang=en> and <https://d-maps.com/carte.php?num_car=4976&lang=en>.

The terms of use for these maps are found here: <https://d-maps.com/conditions.php?lang=en>

7) We note that your Data Availability Statement is currently as follows: "All relevant data are within the manuscript and its Supporting Information files". Please confirm at this time whether or not your submission contains all raw data required to replicate the results of your study. Authors must share the “minimal data set” for their submission. PLOS defines the minimal data set to consist of the data required to replicate all study findings reported in the article, as well as related metadata and methods (<https://eur01.safelinks.protection.outlook.com/?url=https%3A%2F%2Fjournals.plos.org%2Fplosone%2Fs%2Fdata-availability%23loc-minimal-data-set-definition&data=05%7C02%7Cwim.dekesel%40uantwerpen.be%7Cd8e5ebfb398949f19e4308dcb08f7f8d%7C792e08fb2d544a8eaf72202548136ef6%7C0%7C0%7C638579377569332752%7CUnknown%7CTWFpbGZsb3d8eyJWIjoiMC4wLjAwMDAiLCJQIjoiV2luMzIiLCJBTiI6Ik1haWwiLCJXVCI6Mn0%3D%7C0%7C%7C%7C&sdata=%2B6NReuVktOrkukMfRnIClk4Z%2BWyWgqONEgIJVDqv9%2Fg%3D&reserved=0>).
For example, authors should submit the following data:
1) The values behind the means, standard deviations and other measures reported;
2) The values used to build graphs;
3) The points extracted from images for analysis..
Authors do not need to submit their entire data set if only a portion of the data was used in the reported study.
If your submission does not contain these data, please either upload them as Supporting Information files or deposit them to a stable, public repository and provide us with the relevant URLs, DOIs, or accession numbers. For a list of recommended repositories, please see <https://eur01.safelinks.protection.outlook.com/?url=https%3A%2F%2Fjournals.plos.org%2Fplosone%2Fs%2Frecommended-repositories&data=05%7C02%7Cwim.dekesel%40uantwerpen.be%7Cd8e5ebfb398949f19e4308dcb08f7f8d%7C792e08fb2d544a8eaf72202548136ef6%7C0%7C0%7C638579377569336762%7CUnknown%7CTWFpbGZsb3d8eyJWIjoiMC4wLjAwMDAiLCJQIjoiV2luMzIiLCJBTiI6Ik1haWwiLCJXVCI6Mn0%3D%7C0%7C%7C%7C&sdata=FDgc4v5vdLN2PhRqpSrFqTxiqfnNbVT8HzjIRb9YKvk%3D&reserved=0>.
If there are ethical or legal restrictions on sharing a de-identified data set, please explain them in detail (e.g., data contain potentially sensitive information, data are owned by a third-party organization, etc.) and who has imposed them (e.g., an ethics committee). Please also provide contact information for a data access committee, ethics committee, or other institutional body to which data requests may be sent. If data are owned by a third party, please indicate how others may request data access.

All relevant data are within the manuscript and its Supporting Information files.

8) Please amend your detailed Financial Disclosure statement. This is published with the article. It must therefore be completed in full sentences and contain the exact wording you wish to be published.
1) State what role the funders took in the study. If the funders had no role in your study, please state: "The funders had no role in study design, data collection and analysis, decision to publish, or preparation of the manuscript."
2) If any authors received a salary from any of your funders, please state which authors and which funders..
If you did not receive any funding for this study, please simply state: u201cThe authors received no specific funding for this work.u201d

This study was funded by The Research Foundation – Flanders (FWO) through the Senior research project G054820N (to KKA, EV and MP) and PhD fellowship 1171023N (to WDK) (https://www.fwo.be/en/).

WDK received a salary from the Senior research project G054820N for the year 2020-2021 and is currently paid from the PhD fellowship 1171023N. The funders had no role in study design, data collection and analysis, decision to publish, or preparation of the manuscript.
